# Knee hemarthrosis after arthroscopic surgery in an athlete with low factor XIII activity

**DOI:** 10.1186/1758-2555-4-35

**Published:** 2012-10-02

**Authors:** Akira Tsujii, Yoshinari Tanaka, Yasukazu Yonetani, Yoshiki Shiozaki, Yoshiaki Tomiyama, Shuji Horibe

**Affiliations:** 1 Department of Sports Orthopaedics, Osaka Rosai Hospital, 1179 Nagasone-cho, Kita-ku, Sakai, Osaka, 583-8555, Japan; 2 Department of Orthopaedic Surgery, Seifu Hospital, Sakai, Japan; 3 Department of Blood Transfusion, Osaka University Hospital, Suita, Osaka, Japan; 4 Graduate School of Comprehensive Rehabilitation, Osaka Prefecture University, Habikino, Osaka, Japan

## Abstract

We report a thirteen-year-old tennis player with knee hemarthrosis caused by low factor XIII activity. She visited our hospital because of medial peripatellar pain for two years. Although there was no abnormal sign in X-ray or MRI, diagnostic arthroscopy was performed. It revealed some cartilage debris, medial plica and complete septum of suprapatellar plica. Removing the debris by washing out and resecting the medial plica, she could return to play tennis without perioperative symptom. Two months after the first operation, her knee got swelling without any apparent cause. Since 20 ml blood was aspirated twice and MRI revealed suprapatellar mass, we performed arthroscopy again. Suprapatellar mass was old blood clot covered with complete suprapatellar plica. Resection of suprapatellar plica and washing out blood clot were performed, and severe postoperative hemarthrosis was progressively occurred. As factor XIII level was 54% preoperatively, we diagnosed that this condition was caused by low activity level of the factor and administered factor XIII concentrates. The level got improved to 129% and then hemarthrosis gradually relieved. She had no signs of recurrence. We should keep in mind of low factor XIII activity case in case of unexplained postoperative hemarthrosis after arthroscopy because consumption of the factor might promote this condition.

## Background

Hemarthrosis is one of the most common complications after knee arthroscopy [1]. Hemarthrosis is generally not severe except in coagulation factor deficiency. In case of hemophilia this complication is critical, and standard coagulation studies can reveal this disorder. But deficiency of factor XIII cannot be suggested with those studies. Therefore, it is necessary to test factor XIII activity or clot solubility test for diagnosing this disorder. Factor XIII is known as fibrin stabilizing factor, which acts in the terminal phase of the coagulation cascade. The disorder presents clinically with bleeding diathesis, and impaired wound healing. Congenital deficiency of factor XIII was first described in 1960 by Duckert [2] and acquired deficiency of the factor was also reported in some articles [3-5]. However, there are few literatures associated with deficiency of factor XIII especially in the field of orthopedic surgery [6]. We here present a case of postoperative hemarthrosis caused by low activity level of this factor.

## Case presentation

**Figure 1:**
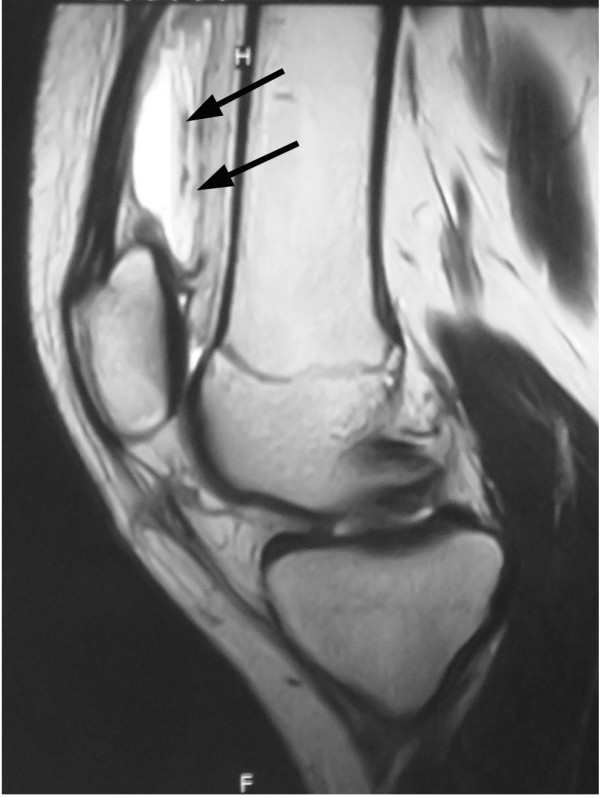
**T2 weighted MR image shows high intensity mass in the suprapatellar pouch (arrows)**.

**Figure 2:**
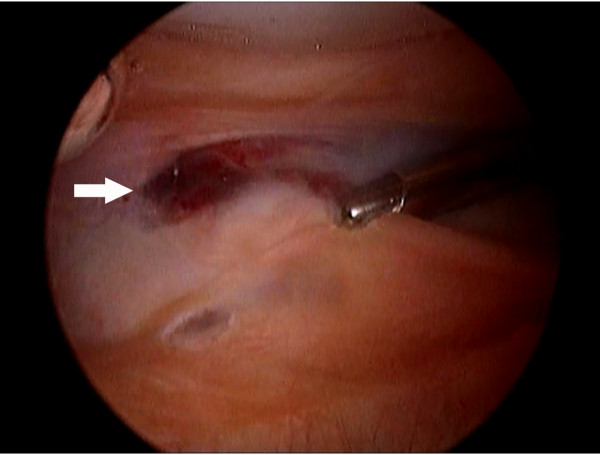
**Bloody content flowed out from suprapatellar pouch (arrow)**.

A thirteen-year-old junior tennis player, who had had no past history of disorder and played several hours a day since 6 years old, visited our hospital with the chief complaint of left knee pain after tennis games for two years. She had a medial peripatellar pain and knee flexion angle was slightly restricted. Although X-ray and MRI revealed no abnormal findings, we performed diagnostic arthroscopy because she and her parents had strong desire to receive arthroscopic inspection. Arthroscopy showed that the ACL and menisci were intact, but there were some cartilage debris. In addition, there were the medial plica and the suprapatellar plica (plica synovialis suprapatellaris, PSSP) with a complete septum, then the debris was washed out and the medial plica was resected. Her knee flexion angle got improved postoperatively, followed by return to the tennis court. Two months after the operation, her left knee got swelling after a long distance walk. Approximately 20 ml of bloody effusion was aspirated by joint puncture twice, and after this procedure the knee swelling increased. T2 weighted MR images revealed high intensity mass in the suprapatellar pouch, indicating of hematoma (Figure 1). As knee flexion angle was gradually restricted again to 110^o^, we decided to perform arthroscopic removal of the hematoma. In order to investigate the cause of unexplained hematoma, we examined factor XIII preoperatively and the level was 54% which was slightly low (normal; over 70%), with no abnormal findings in the standard coagulation studies. At arthroscopy, there was no bloody effusion or hemosiderin deposit within the joint, but in the proximal side of PSSP complete septum there seemed to be blood like content. Resecting the septum with shaver, old blood clot was flowed out (Figure 2). Complete resection of the septum and old blood clot was performed as possible. On the next day, there was no bleeding from the wound and total drainage volume was 90 ml, then we removed the drainage tube. However, her knee got swelling progressively and the flexion angle was severely restricted to 30^o^. Hemoglobin was decreased from 13.5 g/dl preoperatively to 10.3 g/dl on postoperative days (POD) 7 and blood loss estimated to about 800 ml [7]. After discussion with a hematologist, we diagnosed that this condition was caused by low activity level of factor XIII and administered factor XIII concentrates on POD 7 to 9. The level got improved to 129% and then the knee swelling gradually relieved (Table 1). After 3 months postoperatively, she returned to play tennis and after 5 months she completely returned to the game. There was no recurrence but the level of factor XIII returned to preoperative level (57%), which was slight low, three months after the second operation.

**Table 1:** Changes of Hemoglobin (g/dl) and XIII activity (%)

	**Pre**	**POD 7**	**POD 8**	**POD 9**	**POD 13**	**PO 3mo**
Hemoglobin (g/dl)	13.5	10.3	10.8	10.6	10.8	13.1
XIII activity (%)	54	-	98	129	-	57

* Factor XIII concentrates administered on postoperative days (POD) 7, 8, and 9.

## Discussion

We presented a case of severe knee hemarthrosis after knee arthroscopy. Although hemarthrosis is one of the most common complications after knee arthroscopy [1], it is generally not severe and often relieved spontaneously, except in coagulation factor deficiency. In case of hemophilia, which deficit factor VIII or IX, this complication is critical, and preoperative standard coagulation studies can reveal this disorder. But deficiency of factor XIII cannot be suggested with the standard coagulation examination, including prothrombin time, partial prothrombin time, thrombin time, bleeding time, platelet count, and qualitative platelet function assays. Therefore, it is necessary to test factor XIII activity or clot solubility test for diagnosing this condition.

Factor XIII, also known as fibrin stabilizing factor, is an enzyme required for normal fibrin clot formation. It acts in the terminal phase of the coagulation cascade, after thrombin has converted fibrinogen to fibrin. In the absence of factor XIII a clot is easily soluble, so its deficiency leads to prolonged bleeding which is characteristically delayed 12–36 h after stop bleeding. Congenital deficiency of factor XIII was first described in 1960 by Duckert [2]. The disorder presents clinically with bleeding diathesis, and impaired wound healing. And it is also reported about acquired deficiency of factor XIII, caused by formation of antibody or unknown reason [3-5]. According to the European data, the most common bleeding symptoms were subcutaneous bleeding, muscle hematoma, hemorrhage after surgery, hemarthrosis, and intracerebral bleeding [8]. Decreased enzymatic activity of factor XIII was discussed as a cause of unexplained intraoperative and/or postoperative hematoma in abdominal, gynecological, plastic, and urological surgery and also recently in the field of neurosurgery [9]. To the best of our knowledge, however, there were few literatures about periarticular hematoma due to factor XIII deficiency or decreased activity level of factor XIII during perioperative phase. In this present case, low activity level of the factor seemed to be the cause of unexplained bleeding. As its concentration was recovered by administering the factor XIII concentrates, it was highly unlikely that the condition was associated to autoimmune antibody. In addition to the preoperative low activity level, consumption of the factor was likely to aggravate bleeding diathesis. As described by Korte, acquired factor XIII deficiency in the perioperative setting might be frequent [10]. Furthermore, Spahn demonstrated that blood loss could be reduced with administration of a single dose of factor XIII given within 15 min commencement of surgery [11].

As for perioperative level of factor XIII, no report has clearly revealed when we should administer factor XIII concentrates. In general, it is considered sufficient to keep 10% in small bleed, or 20-30% in muscle hematoma. But in severe bleeding case or major surgery, increasing use of the factor in the wound, it is recommended to keep 100% preoperatively [12]. Gerlach et al. reported the average postoperative decrease in factor XIII was approximately 18% in the neurosurgery patients [9]. Furthermore, they concluded the risk of postoperative hematoma is increased in patients with factor XIII < 60%. Taking the conclusion into account, the perioperative low activity level of factor XIII might have been the risk of the hemarthrosis following arthroscopy.

## Conclusion

We report a case of postoperative hemarthrosis caused by low factor XIII activity. Decreased activity of the factor seemed to be the cause, and the consumption might have an effect on bleeding diathesis. We should be in careful about managing this condition perioperatively as coagulation factor activity can decrease by increasing consumption of the factor.

## Consent

Written informed consent was obtained from the patient for publication this case report and the images.

## Competing interests

The authors declare that they have no competing interests.

## Authors’ contributions

All authors co-wrote the paper and discussed the results for the manuscript preparation. All authors have read and approved the final manuscript.

## References

[B1] Small NC Complications in arthroscopic surgery performed by experienced arthroscopists Arthroscopy 1988 4 215 221 10.1016/S0749-8063(88)80030-6 3166663

[B2] Duckert F Jung E Sherling DH A hitherto undescribed congenital haemorrhagic diathesis probably due to fibrin stabilizing factor deficiency Thromb Diath Haemorrh 1960 5 179 186 13724728

[B3] Gregory TF Cooper B Case report of an acquired factor XIII inhibitor: diagnosis and management Proc (Bayl Univ Med Cent) 2006 19 221 223 17252037 10.1080/08998280.2006.11928166 PMC1484527

[B4] Lim W Moffat K Hayward CP Prophylactic and perioperative replacement therapy for acquired factor XIII deficiency J Thromb Haemost 2004 2 1017 1019 10.1111/j.1538-7836.2004.00728.x 15140148

[B5] Ajzner E Schlammadinger A Kerényi A Bereczky Z Katona E Haramura G Boda Z Muszbek L Severe bleeding complications caused by an autoantibody against the B subunit of plasma factor XIII: a novel form of acquired factor XIII deficiency Blood 2009 113 723 725 10.1182/blood-2008-09-179333 18955560

[B6] Thakker S McGehee W Quismorio FP Jr Arthropathy associated with factor XIII deficiency Arthritis Rheum 1986 29 808 811 10.1002/art.1780290617 3718569

[B7] Johansson T Lisander B Ivarsson I Mild hypothermia does not increase blood loss during total hip arthroplasty Acta Anesthesiol Scand 1999 43 1005 1010 10.1034/j.1399-6576.1999.431006.x 10593462

[B8] Ivaskevicius V Seitz R Kohler HP Schroeder V Muszbek L Ariens RA Seifried E Oldenburg J Study Group International registry on factor XIII deficiency: a basis formed mostly on European data Thromb Haemost 2007 97 914 921 17549292

[B9] Gerlach R Tölle F Raabe A Zimmermann M Siegemund A Seifert V Increased risk for postoperative hemorrhage after intracranial surgery in patients with decreased factor XIII activity: implications of a prospective study Stroke 2002 33 1618 1623 10.1161/01.STR.0000017219.83330.FF 12053001

[B10] Korte W Factor XIII in preoperative coagulation management Best Pract Res Clin Anaesthesiol 2010 24 85 93 10.1016/j.bpa.2009.09.011 20402172

[B11] Spahn DR Asmis LM Excessive perioperative bleeding: are fibrin monomers and factor XIII the missing link? Anesthesiology 2009 110 212 213 19194145 10.1097/ALN.0b013e3181942c65

[B12] Fukue H Arai M Factor XIII A subunit deficiency The Journal of Japanese Society on Thrombosis and Hemostasis 2001 12 66 73 10.2491/jjsth.12.66

